# Exclusion and Underdetermined Qualia

**DOI:** 10.3390/e21040405

**Published:** 2019-04-16

**Authors:** Kyumin Moon

**Affiliations:** Department of Philosophy, Seoul National University, Seoul 151-742, Korea; dkxnaks@snu.ac.kr

**Keywords:** integrated information theory, consciousness, qualia, experience, phenomenology, the qualia underdetermination

## Abstract

Integrated information theory (IIT) asserts that both the level and the quality of consciousness can be explained by the ability of physical systems to integrate information. Although the scientific content and empirical prospects of IIT have attracted interest, this paper focuses on another aspect of IIT, its unique theoretical structure, which relates the phenomenological axioms with the ontological postulates. In particular, the relationship between the exclusion axiom and the exclusion postulate is unclear. Moreover, the exclusion postulate leads to a serious problem in IIT: the quale underdetermination problem. Therefore, in this paper, I will explore answers to the following three questions: (1) how does the exclusion axiom lead to the exclusion postulate? (2) How does the exclusion postulate cause the qualia underdetermination problem? (3) Is there a solution to this problem? I will provide proposals and arguments for each question. If successful, IIT can be confirmed with respect to, not only its theoretical foundation, but also its practical application.

## 1. Introduction

Integrated information theory (IIT) states that the brain generates conscious experience by virtue of its capacity to integrate information. Identifying consciousness with integrated information, IIT attempts to explain both the levels and the qualities of experience in a mathematical manner. Levels of consciousness can be quantitatively measured by the values of Φ. Qualities of consciousness can be geometrically represented in a multidimensional space termed the concept space [1,2,3]. From its initial version to the most recent version, IIT has been updated to provide a principled and theoretical framework for the scientific account of consciousness. Currently, a growing number of researchers in various fields, including neuroscience, psychiatry, computer science, and physics, have shown significant interest in the theory.

IIT intends to explain neural data regarding consciousness in a unified manner. It also predicts that artificial consciousness is possible: “[T]o the extent that a mechanism is capable of generating integrated information, no matter whether it is organic or not, … and independent of its ability to report, it will have consciousness” [1]. Although these scientific contents and empirical prospects of IIT have received much attention, the theoretical structure of IIT has attracted relatively less interest. IIT starts by setting out two sets of a priori theses that constrain and guide the theory [3,4]. The first set is labeled *phenomenological axioms*. These axioms describe a number of the fundamental properties of consciousness. The second is termed *ontological postulates*, describing how physical systems should be in order to produce consciousness. These postulates are implemented by mathematical models and technical concepts in IIT. Compared with other hypotheses in the field, this ‘axiomatic approach’ to consciousness is truly distinctive of IIT [5]. Interestingly, while there is a strong one-to-one correspondence between the axioms and postulates, a specific link from each axiom to each postulate has not been elucidated thus far.

With regard to the specific modeling of IIT, one of the postulates causes a serious problem. The exclusion postulate holds that the maximally integrated cause and effect (MICE) repertoire must be chosen as ‘quale sensu stricto’ (quale in the narrow sense), and others should be excluded. Although ‘MICE’ is also known as a concept, to avoid possible terminological confusion, I will use the term ‘MICE’ only [6] (p. 16, Footnote 18). Overall, MICE can be thought of as the ‘building blocks’ of consciousness, which constitute an experience itself. This notion of MICE seems fairly similar or even identical to what philosophers call ‘qualia’, the phenomenal quality of conscious experience. The current model of IIT, however, allows that there can be several different MICEs. When this occurs, IIT cannot identify the genuine quale. This issue of finding the real quale from multiple MICEs was systematically raised for the first time by Krohn and Ostwald under the title “quale underdetermination” [7] (p. 15).

In this paper, I will attempt to answer the following three questions: (1) how does the exclusion axiom lead to the exclusion postulate? (2) How does the exclusion postulate cause the problem of underdetermined qualia? Finally, (3) is there a solution to this problem? As the exclusion postulate is closely related to the exclusion axiom, in order to assess the qualia underdetermination problem, one should start by examining the relationship between the exclusion axiom and the postulate. First, one possible method of inducing the exclusion postulate from the exclusion axiom will be suggested (Section 2). Second, the problem of underdetermined qualia will be described in detail. Examining a couple of the solutions suggested to date, a novel solution will be suggested (Section 3). Third, by interpreting the fundamental ideas of IIT more thoroughly, I shall argue for the proposed novel solution to the qualia underdetermination problem (Section 4). If the arguments in this paper are successful, IIT can be confirmed with regard to not only its theoretical foundation but also its mathematical modeling.

## 2. From Phenomenology to Ontology: How the Exclusion Axiom Leads to the Exclusion Postulate

IIT has many interesting and even seemingly problematic predictions, such as sophisticated panpsychism, the possibility of zombie systems, fading and dancing qualia, and the paradox of certainty [6,8,9,10,11,12,13,14,15,16]. Despite these controversial issues, the intellectual fervor toward IIT has never withered. Recently, theoretically fundamental aspects of IIT have been examined and articulated. For instance, the axiomatic approach of IIT has been critically examined [5]. In addition, several methods by which to articulate the notion of information in IIT have been suggested [17,18,19]. Further, many efforts have been made with regard to empirical applications of IIT. Some suggest more efficient ways to identify the minimal informational partition (MIP) [20,21]. Others develop practical alternative measures for the integration of information [22,23,24]. Indeed, theoretically and empirically, IIT is making progress.

However, it appears that the theoretical structure of IIT receives relatively little attention. IIT, at least its current version, starts with a number of axioms and postulates: five phenomenological axioms regarding consciousness and five ontological postulates regarding the physical substrates of consciousness. Technically, with the exception of the first two postulates, existence and composition, the postulates are divided into two levels: a level of mechanisms and a level of systems of mechanisms. This axiomatic approach is clearly unique. To the best of my knowledge, no scientific theories of consciousness follow such an approach.

Justifying each axiom and postulate is not the aim of this paper. My focus is rather on the relationships between them. Given that the main topic of this paper is the qualia underdetermination problem, which primarily involves mechanisms, I will only consider postulates regarding mechanisms. The parallel structure between axioms and postulates strongly suggests that there are certain intimate relationships among them. The authors of IIT, however, merely state the following: “To parallel the phenomenological axioms, IIT posits a set of postulates” [3] (p. 3). Although axioms and postulates are fundamental to the theory, it is unknown why postulates correspond to axioms. That is, even when one accepts the axioms regarding experience, she can legitimately raise the question of why she must accept the postulates regarding mechanisms that contribute to experience. Simply put, how can we *induce* or *derive* the postulates regarding the physical mechanisms of consciousness from the axioms regarding consciousness [6] (pp. 11–12)? How does phenomenology constrain ontology?

While the exact relationships between axioms and postulates are unclear, for some of them, we can easily infer such relationships. For instance, how can one derive the information postulate from the information axiom? The information axiom states that consciousness is informative. That is, “each experience differs in its particular way from other possible experiences” [3] (pp. 2–3). If experience is essentially informative and a mechanism contributes to experience, such a mechanism must also be informative. How can something uninformative contribute to something essentially informative? The natural way for mechanisms to be informative is by producing information. Thus, we have the information postulate: “A mechanism can contribute to consciousness only if it specifies ‘differences that make a difference’ within a system. That is, a mechanism in a state generates information only if it constrains the states of a system that can be its possible causes and effects—Its cause-effect repertoire” [1] (p. 3).

With the integration axiom and postulate, one can take similar steps. The exclusion axiom states that consciousness is integrated, meaning that “each experience is (strongly) irreducible to non-interdependent components” [3] (p. 3). If experience is fundamentally integrated, in order to contribute to such experience, the mechanisms should generate integration or irreducibility. One method to generate integration or irreducibility is by producing integrated information. Thus, we have the integration postulate. “A mechanism can contribute to consciousness only if it specifies a cause-effect repertoire (information) that is irreducible to independent components” [3] (p. 3). It appears that, at least for information and integration, one can plausibly or at least intuitively infer the postulates from the axioms.

Given the analysis above, we can expect that there are plausible or intuitive derivations from the axioms to postulates. Of course, these derivations cannot be the formally rigorous deductions found in mathematics or logic. In mathematics or logic, one can deduce theorems from axioms by applying a limited number of inferential rules and previously proven theorems. Derivations between the phenomenological axioms and ontological postulates, however, are neither mathematical proofs nor logical derivations. They are apparently plausible or intuitive inferences. Although the terms “axioms” and “postulates” prompt us unwittingly to expect formally rigorous proofs or deductions, it appears that we cannot logically or mathematically deduce the postulates from the axioms. The best we can do is to establish plausible or intuitive inferential relationships between them.

At this point, the question remains as to whether we plausibly or intuitively induce the exclusion postulates from the exclusion axiom. The exclusion axiom states that consciousness is exclusive. “[E]ach experience excludes all others–at any given time there is only one experience having its full content, rather than a superposition of multiple partial experiences; each experience has definite borders–certain things can be experienced and others cannot” [3] (p. 3). The point is clear: experiences cannot be superposed. Overlapping consciousness is impossible. Hence, one may consider that if experience is essentially exclusive, in order for a mechanism to contribute to such exclusive experience, it must exclude all cause–effect repertoires aside from the maximally irreducible one. This inference leads to the exclusion postulate: “A mechanism can contribute to consciousness at most one cause-effect repertoire, the one having the maximum value of integration/irreducibility Φ^Max^. This is its maximally irreducible cause-effect repertoire (MICE, or quale sensu stricto)” [3] (p. 3).

Compared with cases of information and integration, however, it is not clear as to whether the exclusion postulate can be plausibly or intuitively derived from the exclusion axiom. The problem is that the exclusivity of experience does not appear to require the exclusive, unique integrated information generated by mechanisms. Even when a mechanism produces multiple pieces of integrated information and excludes nothing, it remains possible for experience to be exclusive and not superposed. This contrasts with cases of information or integration. For instance, in order for experience to be informative, the mechanisms contributing to experience must produce information. If the mechanisms do not produce information, there seems to be no explanation as to why experience is informative. However, if experience is fundamentally integrated and a mechanism contributes to experience, it seems necessary that a mechanism integrates information. Producing integrated information appears to be the only explanation as to why experience is essentially irreducible to its independent parts. In these cases, we can plausibly or naturally infer the information or integration postulate from the corresponding axioms. Nonetheless, in the case of exclusion, this inference seems less plausible. How the exclusion axiom leads to the exclusion postulate or how the exclusion of non-maximal integrated information explains the exclusivity of experience is not clear.

One may note that IIT has already provided a reason for the exclusion postulate. It appears that the central rationale is derived from “a principle of causal parsimony, or a causal version of Occam’s razor, roughly saying ‘do not unnecessarily multiply causes’” [3] (pp. 9–10) (see also [4] (pp. 301–302)). Oizumi et al. seem to believe that this principle enforces only one cause. However, there is a grounding factor of IIT: to exist as a whole is to make differences beyond and above the parts of the whole, i.e., to be irreducible [3]. The most irreducible cause would then make the most difference over and above its parts to a mechanism in a state. In such a case, although the most irreducible cause can be said to exist as a whole, other less irreducible causes cannot. Given the most irreducible cause, even if less irreducible causes are decomposed, the differences they would have made would be made by the most irreducible one. Regardless of whether or not the less irreducible causes exist as a whole, every difference will be made by the most irreducible cause. In this sense, the less irreducible causes cannot make any difference and fail to exist. If a mechanism must have only one cause, and only the most irreducible cause can exist, then only cause must be maximally irreducible. The same applies to effects. Therefore, we have the exclusion postulate: a mechanism that contributes to experience can have only MICE.

The arguments above justify only half of the exclusion postulate. The exclusion postulate has two parts; first, it states that a mechanism can contribute to experience by specifying only one irreducible cause–effect repertoire. Second, the postulate claims that the only repertoire must be maximally irreducible. The fundamental idea of IIT may explain the second part of the exclusion postulate. One may understand why the unique irreducible cause–effect repertoire must be the one that is maximally irreducible. Even so, relying on Occam’s razor does not explain the first part. Occam’s razor requires that causes be as minimal as possible. However, being minimal does not necessarily mean being unique. Even if one does not unnecessarily multiply causes, there may still be two or more causes. It is unclear how the exclusion axiom yields the conclusion that the irreducible cause–effect repertoire specified by a mechanism contributing to experience must be unique, but this is the question to be answered. How does the uniqueness of experience result in the uniqueness of the irreducible cause-effect repertoire? Unless this question is answered, one can legitimately require further justification for drawing the exclusion postulate from the exclusion axiom.

Thus, I argue that there is a possible means of plausibly or intuitively inducing the exclusion postulate from the exclusion axiom. The inference is as follows:

(1) Experience is exclusive (the exclusion axiom).

(2) If a mechanism contributes to experience by specifying multiple irreducible cause–effect repertoires, then it contributes to one or several experiences.

(3) If a mechanism contributes to one experience by specifying multiple irreducible cause–effect repertoires, then the experience cannot be exclusive.

(4) If a mechanism contributes to several experiences by specifying multiple irreducible repertoires, then those experiences cannot be exclusive.

(5) A mechanism contributes to experience by specifying a unique irreducible cause–effect repertoire.

(6) A unique irreducible cause–effect repertoire specified by a mechanism must be maximally irreducible. That is, it must be MICE.

(7) A mechanism can contribute to experience only by specifying MICE (the exclusion postulate).

Premise (1) is the exclusion axiom. Premise (2) is logically apparent. If Premise (6) is true, then we have the exclusion postulate as a conclusion. The crucial part is the inference from Premise (3) to Premise (6). Below, I will explain each premise in turn.

According to Premise (3), an experience cannot be exclusive when a mechanism contributing to the experience specifies multiple irreducible cause–effect repertoires. If a mechanism contributes to one experience by specifying multiple irreducible cause–effect repertoires, the mechanism would contribute to the experience multiple times simultaneously. Because the mechanism’s contribution to the experience would be reflected by a certain part of the experience, the natural consequence is that the part of the experience is somehow superposed. For instance, suppose that a mechanism contributes to Mary’s visual experience by specifying two irreducible cause–effect repertoires. As the two repertoires are different, each contributes to her visual experience in different ways. One repertoire may contribute to Mary’s phenomenal visual field by producing a red quale. Another may contribute to her visual field by generating a blue quale. As a result, somewhere in Mary’s phenomenal visual field, the red quale and blue quale will overlap. That is, Mary’s visual experience should be partially superposed. This partial superposition in experience by overlapping qualia violates the exclusion axiom. Therefore, when a mechanism contributes to a single experience by specifying multiple irreducible cause–effect repertoires, the experience cannot be exclusive.

Some may argue that two different irreducible cause–effect repertoires may not produce two different qualia. Rather, it seems possible that two repertoires conjointly generate a single quale. In the case mentioned above, for example, two repertoires can generate one quale, which may be a purple quale or a neither-red-nor-blue quale. While this scenario appears to be feasible at first glance, there is a good reason to believe that it cannot be the case. In the framework of IIT, specifying two different irreducible cause–effect repertoires means producing two different pieces of integrated cause–effect information. If two irreducible cause–effect repertoires somehow produce one quale, the two pieces of integrated information must be ‘merged’ into one. In IIT, the only means by which this merging could occur is integration. Thus, in order to generate a single quale by specifying two repertoires, a mechanism must produce two pieces of integrated information and integrate them once again. The problem is that integrating integrated information cannot occur via a single mechanism. According to IIT, integration of information occurs among multiple different mechanisms, not within a single mechanism. “Clearly, for integrated information to be high, a system must be connected in such a way that information is generated by causal interactions among rather than within its parts” [3] (pp. 220–221). Hence, there is no means by which a mechanism could produce a single quale by specifying multiple irreducible cause–effect repertoires.

Premise (4) states that experience cannot be exclusive when a mechanism contributes to multiple experiences by specifying multiple irreducible cause–effect repertoires. I argued in the previous paragraph that when a mechanism contributes to one experience by specifying multiple repertoires, such an experience must be partially superposed. Accordingly, one may consider whether a mechanism contributes to several experiences. For example, suppose that Mary’s visual field is divided into two color experiences. The right half of her visual field is filled with blue, and the left half is filled with red. One mechanism contributes to both visual fields by specifying two repertoires, as before. In this case, the mechanism contributes to Mary’s two visual fields simultaneously, specifying one repertoire for the blue-right field and another repertoire for the red-left field. Then, Mary’s two visual fields would be partially superposed in that they share a physical substrate. To illustrate this point, consider the case wherein the same neuronal group contributes to two experiences: a visual experience of colors and an auditory experience of sounds. What is naturally expected is synesthesia, i.e., hearing colors or seeing sounds. That is, if both visual and auditory experiences partially share their physical substrates, the two experiences will overlap. This can be generalized regardless of the sensory modality. If the physical substrates of Mary’s blue-right field and red-left field are partially overlapped, the two visual fields themselves must be partially overlapped. Again, this is a violation of the exclusion axiom. Even when a mechanism contributes to multiple experiences by specifying multiple irreducible cause–effect repertoires, those experiences cannot be exclusive.

Premise (5) states that a mechanism contributes to experience by specifying a unique irreducible cause–effect repertoire. The inference from Premise (4) to (5) is apparent. Experience must be exclusive according to Premise (1). However, Premise (4) states that if a mechanism contributes to multiple experiences by specifying multiple irreducible cause–effect repertoires, experience cannot be exclusive. Hence, one must conclude that once a mechanism contributes to experience, it must do so only by specifying a unique irreducible cause–effect repertoire.

Premise (6) argues that the unique irreducible cause–effect repertoire that contributes to experience must be maximally irreducible. We already have discussed the arguments supporting this claim. Irreducible cause repertoires represent irreducible sets of possible causes, and one of the grounding features of IIT is that to exist as a whole is to make differences that are irreducible to the parts of the whole. Only the most irreducible set of causes makes the most irreducible differences in a mechanism. No causal work is left for the less irreducible ones such that they can be said not to exist. Only the most irreducible one can be said to exist. One can treat effect repertoires in the same manner. If a mechanism must have a unique irreducible cause–effect repertoire to contribute to experience and only MICE can exist, the unique irreducible cause–effect repertoire must be MICE.

To recapitulate, the argument from Premise (3) to (6) is driven by reductio ad absurdum: if the exclusion postulate is incorrect, the exclusion axiom cannot be true, and the axiom must be true. Therefore, the postulate also should be true. If a mechanism contributes to experience by specifying multiple irreducible cause–effect repertoires, the experience cannot be exclusive. When multiple maximally irreducible repertoires contribute to a single phenomenological content of conscious experience, the content must be superposed. Even when multiple maximally irreducible repertoires contribute to multiple phenomenological qualities of experience, those qualities must overlap. Either way, experience must defy the exclusion axioms. Henceforth, if the exclusion axiom is maintained, the maximally irreducible cause–effect repertoires contributing to experience must be unique. Hence, the exclusion postulate must be true.

## 3. The Qualia Underdetermination Problem: How Qualia Can Be Underdetermined

In the previous section, I argued that we have good reason to believe that a unique irreducible cause–effect repertoire must be MICE. That is, being unique entails being maximally irreducible. Does the reverse hold? Apparently, it does not. Although being unique entails being maximally irreducible, there is no guarantee that maximum irreducibility entails uniqueness. That is, MICEs can be non-unique, and several can exist. A unique irreducible cause–effect repertoire must be the one that is MICE. However, MICE need not be unique at all. Furthermore, this asymmetry raises a serious problem in the framework of IIT 3.0, known as the qualia underdetermination problem.

MICE involves almost all features of IIT. First, conceptual information (CI) is calculated as the Earth’s mover distance (EMD) between the maximum entropy distributions and MICE [3]. Integrated conceptual information, Φ, also rests on EMD from the maximum entropy distribution to MICE [3]. Accordingly, the maximum value of Φ, Φ^max^, must be dependent on MICE. In IIT, Φ^max^ represents the level of consciousness. Further, MICE is represented as points in the multidimensional space termed the concept space that constitute a ‘constellation’ referred to as the *maximally integrated conceptual structure (MICS)* [3]. IIT 3.0 refers to MICS as ‘quale sensu lato’ (qualia in the broad sense) and directly identifies it with experience itself. The geometrical shape of the MICS represents the quality of consciousness. MICE is so crucial that without it, IIT would collapse into nothing.

Accordingly, the possibility of non-unique MICEs immediately provokes serious problems in all respects mentioned above. First and foremost, it affects theoretically central concepts in IIT: how can we know which MICE is the genuine one? How can we select one MICE to represent the real quality of our consciousness? Given that the CI, Φ, Φ^max^, and MICS change depending on which MICE one selects, when MICE is underdetermined, so are the CI, Φ, Φ^max^, and MICS. If MICE is underdetermined, both Φ^max^ and the shape of the MICS also are underdetermined. Moreover, when multiple MICEs are calculated, one cannot know which MICE is the genuine quale sensu stricto nor which MICS is the real quale sensu lato. In the framework of IIT, these underdeterminations directly imply practical limitations: how can we measure the levels of consciousness? How can we geometrically describe the qualities of consciousness? Once MICE is underdetermined, theoretically as well as practically, everything is underdetermined. The qualia underdetermination problem shakes IIT to the ground.

Krohn and Ostwald provide a good example of underdetermined qualia [7]. Focusing on maximally irreducible cause repertoires, they illustrate a possible case where there are three cause repertoires with the same maximum value of integrated cause information [7] (pp. 12–13). Such repertoires are non-unique maximally irreducible cause repertoires. All three irreducible cause repertoires lead to the same maximal value of integrated cause information over their respective MIPs. In such cases, the correct irreducible cause repertoire is underdetermined. However, the CI of the three cause repertoires will differ from one another, for EMD is sensitive to the actual distributions. In turn, each of three irreducible repertoires will lead to different outcomes for Φ, Φ^max^, and MICS. This case describes how MICE, or qualia, can be multiplied when implementing IIT.

To the best of my knowledge, only two solutions to the qualia underdetermination problem have been proposed. Both concern the dimensionality of repertoires, but each takes the opposite stance. First, Oizumi et al. introduced the highest dimensionality criterion [1]. They argued that when multiple irreducible cause repertoires yielding the same maximum value of integrated cause information are given, one should select that with the highest dimensionality. It is argued that the repertoire with “the largest purview” must be selected (Figure S1 in [3]). As a purview is a means of considering a particular subset of elements of the system, the bigger the purview, the greater the number of system elements accounted for. Thus, Oizumi et al. claim that the highest-dimensional irreducible cause repertoire must be selected, “because it specifies information about more system elements for the same value of irreducibility” (Figure S1 in [3]). Indeed, this is the solution proposed by Tononi in the early stage of IIT. It seems that he was already aware of the problem of underdetermined qualia. He suggests the following possible solution:

If several CER(S) yield the same max, one takes the CER(S) of largest scope (accounting for the most), where ϕ^MIP^(S) > 0, its subsets R have lower or at most equal ϕ^MIP^, and its supersets T have lower ϕ^MIP^: ϕ^MIP^(R) ≤ ϕ^MIP^(S) > ϕ^MIP^ (T), for all R ∈ S and all T ∈ S. If there are multiple maximal CER(S) each with the same scope, then at any given time only one is realized as a concept, although which one is indeterminate [4] (p. 320, Footnote 10).

“Largest scope” in the above quote is analogous to ‘bigger purview’ in the current context. The footnote indicates that the highest dimensionality criterion is not new to IIT. It also outlines that selecting the highest-dimensional MICE cannot be the solution to the qualia underdetermination problem. Even with such a criterion, there can still be a case where “there are multiple maximal CER(S) each with the same scope,” such that the genuine MICE is “indeterminate” [4].

In contrast, Krohn and Ostwald argue for the exact opposite criterion [7]. They suggest the lowest dimensionality criterion. Krohn and Ostwald claim that the principle of causal parsimony or the causal version of Occam’s razor must be strictly interpreted. Occam’s razor requires one to select causes or effects as parsimoniously as possible. According to Krohn and Ostwald, this demand must be met when there are several maximally irreducible cause repertoires. “As such, choosing the distribution (over ‘two causes’) … thus seems less parsimonious than choosing one of the lower-dimensional distributions over fewer causes” [7] (p. 11). This overturns the highest dimensionality criterion: specifying that information regarding more system elements cannot be the criterion for selecting the genuine MICE. Rather, specifying information regarding fewer system elements should be the criterion, because it provides a more parsimonious explanation for all differences of a system in a state.

Selecting the lowest-dimensional MICE, however, is essentially a limited solution to the qualia underdetermination problem. Krohn and Ostwald themselves demonstrated that their own criterion cannot solve this problem. Even when the criterion with the lowest dimensionality is applied, among the three equivalently maximally irreducible cause repertoires, only one can be eliminated. The other two irreducible cause repertoires are equal to one another and therefore remain underdetermined [7] (p. 15). To solve this underdetermination problem, Krohn and Ostwald suggest an additional criterion, which deserves to be called the most shaping criterion: among several MICEs, the one that contributes most to the conceptual structure in unique cases must be selected. The most shaping MICE is the MICE of a particular subset of elements that contributes to most conceptual structures by producing unique concepts. Although this additional criterion can help to solve the given case and makes intuitive sense, Krohn and Ostwald admit that it cannot be generalized [7] (p. 15). Regardless, the qualia underdetermination problem is not easily solved.

Overall, while underdetermined qualia raise a serious problem in IIT, no satisfying solution has been provided. The qualia underdetermination problem occurs when there are multiple MICEs. It affects IIT conceptually as well as practically. The current version of IIT nonetheless has no criterion for selecting one MICE as the genuine quale. The suggested solutions focus on the dimensionality of repertoires. Initially, the criterion with the highest dimensionality was suggested, but this strategy was found to be inadequate. However, the criterion with the lowest dimensionality also cannot cover all possible cases of multiple MICEs, even when it is supplemented by the most shaping criterion. It is highly desirable to find a sensible formal criterion for the selection of MICE.

The failure of previous attempts strongly suggests that accounting for dimensionality or adding further criteria cannot be the solution to the qualia underdetermination problem. It appears that a more principled and a priori approach is required. Merely excluding some MICEs and preserving others would not help. It is tempting to identify a means by which the problem can be prevented. That is, one may attempt to dissolve the qualia underdetermination problem rather than solve it. In the next section, I shall propose a novel criterion and argue that the qualia underdetermination problem can be prevented.

## 4. The Difference-Making Criterion: How Qualia Cannot Be Underdetermined

My suggestion starts by focusing on the notion of integrated information. What does it mean for a mechanism to produce integrated information? Integrated information is information generated by the whole mechanism over and above its parts. When a mechanism generates integrated information, it is “irreducible with the respect of information” [3] (p. 8) (p. 8. See also [25,26]) This irreducibility is related to ‘causal emergence’. [27,28,29] However, “According to IIT, mechanisms that do not generate integrated information do not exist from the intrinsic perspective of a system” [3] (p. 8). If so, integrated information captures the extent to which the mechanism exists as a whole from the perspective of the system itself. That is, how the mechanism irreducibly as well as intrinsically exists is quantitatively measured by φ. In short, integrated information captures the irreducible and intrinsic existence of a mechanism.

Hence, what if a certain mechanism specifies multiple MICEs under different purviews? For the sake of argument, let us focus on maximally irreducible cause repertoires. Suppose that a system consists of the four elements A, B, C, and D. One current mechanism, AB_t_, specifies two MICEs under two purviews: AB_t_/AB_t–1/_ and AB_t_/CD_t−1_. Under both purviews, the φ produced by AB_t_ is 3. Because both past purviews are sufficient to yield the same MICE, this situation can be considered to be a sort of causal overdetermination, the case where there are two or more sufficient causes. One partitions the first purview into AB_t_/[]_t−1_ and []_t_/AB_t−1_ such that the mechanism AB_t−1_ is eliminated. Here, it is important to note that this elimination is not done to calculate how much integrated cause–effect information is generated by AB_t_. The operation of elimination is not performed from the past of the external observer. Rather, it is performed to eliminate the past purview intrinsically. Usually in IIT, one should partition a purview when she needs to calculate integrated information produced from a mechanism as an external observer. In this case, elements outside of the purview in question are marginalized. In the example, however, one may partition the purview AB_t_/AB_t−1_ into its past and present, AB_t_ and AB_t−1_, in order to find out whether AB_t−1_ makes any difference to AB_t_. Thus, no marginalization is needed. Hypothetically, this is a possible operation. From the perspective of the current system ABCD_t_, however, this elimination does not affect in any way the irreducible and intrinsic existence of AB_t_. Even when AB_t−1_ is eliminated, there is still the other purview AB_t_/CD_t−1_, and under this purview, AB_t_ would specify the maximally irreducible cause repertoire and produce the same φ value. Regardless of whether or not AB_t−1_ exists, from the perspective of ABCD_t_, AB_t_ as a whole would still be in its state.

If this is the case, states of AB_t−1_ have no effect on AB_t_ as a whole from the intrinsic perspective of the system. The crucial point is that according to IIT, to exist is to cause differences. “IIT’s information postulate is based on the intuition that, for something to exist, it must make a difference” [1] (p. 10). In order for a certain mechanism to exist from the intrinsic perspective of the system, it must make some intrinsic differences in the system. However, even when AB_t−1_ is eliminated, no intrinsic differences arise. This implies that the states of AB_t–1_ cannot make any intrinsic difference in ABCD_t_. Therefore, the states of AB_t−1_ cannot exist from the intrinsic perspective of ABCD_t_. This means that the repertoires specified by AB_t_ under the purview of AB_t_/AB_t−1_ cannot represent anything that intrinsically exists. In short, the maximally irreducible cause repertoire fails to be a repertoire of something that exists intrinsically.

With regard to the other purview, the situation is the same. Suppose that one partitions the other purview, ABt/CD_t−1_, into AB_t_/[]_t−1_ and []_t_/CD_t−1_. Then, while the past mechanism CD_t−1_ is eliminated, from the intrinsic perspective of ABCD_t_, this would not cause any difference with regard to the irreducible and intrinsic existence of AB_t_ due to the purview AB_t_/AB_t−1_. Under this purview, AB_t_ would generate φ = 3. This implies there is no difference in AB_t_ as a whole from the intrinsic perspective of ABCD_t_. AB_t_ would still be in its state both irreducibly and intrinsically. This absence of difference-making indicates that the states of CD_t−1_ fail to exist from the perspective of ABCD_t_. Again, under the purview of AB_t_/CD_t−1_, the maximally irreducible cause repertoires specified by AB_t_ cannot represent something that intrinsically exists.

If the above analysis is correct, when two or more maximally irreducible cause repertoires are specified by a mechanism, all of them fail to represent something that intrinsically exists. In IIT, the repertoires are probability distributions over past or future states of a certain subset of elements that exist intrinsically. Once multiple maximally irreducible cause repertoires are given, however, the possible states represented by the repertoire cannot be said to exist from the perspective of the system itself. All maximally irreducible cause repertoires fail to be repertoires at all. It is easy to see that the same applies with multiple maximally irreducible effect repertoires. When there are multiple maximally irreducible effect repertoires, these repertoires cannot be said to represent possible effects that exist from the intrinsic perspective of the system. Therefore, when there are non-unique MICEs, paradoxically, all MICEs fail to be repertoires.

The argument thus far suggests a new criterion for the qualia underdetermination problem: given the argument, when there are multiple MICEs, which MICE is the genuine one? The answer is that none of them can be the genuine MICE. The rationale for this answer is that none of the multiple MICEs can be cause or effect repertoires. All multiple MICEs fail to represent something that makes any intrinsic difference in the system. Conversely, for a MICE to be the genuine MICE, it must represent something that makes a difference from the intrinsic perspective of the system. Thus, one could say that only a MICE representing the states of a mechanism that make differences from the perspective of a system itself can be the genuine MICE; i.e., it is the real quale. This criterion deserves to be called the difference-making criterion. According to this criterion, the qualia underdetermination problem does not occur, as there cannot be multiple MICEs in the first place. As soon as there are multiple MICEs, all such MICEs will be excluded as non-repertoires. As mentioned at the end of the previous section, the difference-making criterion does not solve the qualia underdetermination problem. Rather, it dissolves or prevents the problem.

If all multiple MICEs cannot be the real quale, then what should be selected? I believe that one should find ‘the next big thing’ of irreducible cause–effect repertoires. When multiple maximally irreducible cause–effect repertoires are excluded, there would be a cause–effect repertoire that is less irreducible than the maximally irreducible repertoire but more irreducible than others. This next maximally irreducible cause–effect repertoire can be a proper candidate for the genuine MICE. Nonetheless, what if there are multiple next maximally irreducible cause–effect repertoires? For the reasons provided earlier, the multiple ‘next big things’ should be excluded and the lesser irreducible one should be selected as the genuine MICE. Overall, one can exclude all multiple MICEs until she identifies the unique MICE. In this sense, the procedure of identifying the genuine MICE described in this section does not allow the qualia underdetermination problem.

Of course, there are various possibilities that require further investigation. For instance, while all of the examples in this paper implicitly assume deterministic systems under ideal conditions, it is nonetheless possible that under actual conditions, noise and/or spontaneous fluctuations from the external environments of mechanisms may resolve underdetermined qualia. There can also be ‘a top down criterion’: even when there are multiple MICEs, how they contribute to a large phi can differ. Then, one may discern one that contributes to the maximum or minimum among the alternative values of Φ^MAX^.

To summarize, the qualia underdetermination problem can be prevented by adopting a novel criterion for the genuine MICE, in this case the difference-making criterion. Under the difference-making criterion, there cannot be multiple MICEs, as once there are multiple MICEs, none can be repertoires. The only means left is to select the unique MICE. In this sense, the qualia cannot be underdetermined; they must always be uniquely determined.

## 5. Conclusions

While several conceptual and technical limitations have been raised against IIT, to date IIT appears to be one of the leading theories in the field of consciousness studies. Its bold approach to consciousness, which directly identifies experience with regard to MICS, has attracted the attention of researchers from diverse fields. It is true that the explanatory and predictive power of IIT mainly derives from its articulated mathematical model and applicability. The current version of IIT, IIT 3.0, not only explains a wide range of empirical data in an integrative manner but also provides interesting and testable predictions regarding many interesting issues in the science of consciousness. However, the uniqueness of the theoretical structure of IIT has received relatively less attention. The relationship between phenomenological axioms and ontological postulates deserves a deeper analysis. In this paper, focusing on the link from the exclusion axiom to the exclusion postulate, I have provided one possible means by which to understand how the phenomenology of exclusion constrains the ontology of exclusion.

Further, there is a delightful and disturbing problem that originates from the exclusion postulate. Although the postulate enforces that only MICE can be the real quale, when multiple MICEs are specified by a mechanism, IIT cannot determine which is the genuine MICE. Even the most recent version of IIT, IIT 3.0, is affected by this qualia underdetermination problem. Conceptually, the underdetermination of qualia affects other central concepts of IIT, including the Φ, Φ^max^, and MICS concepts. Practically, it prevents us from determining which Φ^max^ is the real level of consciousness and which MICS shape represents the quality of consciousness. A couple of solutions to the qualia underdetermination problem have been suggested, but none were successful in excluding all possible cases of multiple MICEs. To prevent or dissolve the qualia underdetermination problem, I have argued for the difference-making criterion, which does not allow multiple MICEs.

The arguments for the novel criterion are derived from a thorough reading of the fundamental ideas and intuitions in IIT. Therefore, unless there are conceptually radical changes in IIT, the proposals developed in this paper could be applied not only to the current version, but also to any version of IIT. The core concepts of IIT, specifically integrated information and existence, are essentially related to the notions of irreducibility, intrinsicality, and difference-making. Regardless of the detailed differences in the versions or formalisms, any theoretical reading of IIT must consider these notions more seriously. This strict interpretation may shed some light on the inherent problems of IIT.
